# Supplementary material for: Pyruvate Dehydrogenase Kinase 1 inhibition mediated oxidative phosphorylation enhancement in cartilage promotes osteoarthritis progression
Source: BMC Musculoskelet Disord. 2023 Jul 20;24:597. doi: 10.1186/s12891-023-06585-6 (PMC10357736; doi:10.1186/s12891-023-06585-6)
Supplement: Supplementary file 1 — Additional file 1. [file 12891_2023_6585_MOESM1_ESM.zip › KEGG.Gsea.1653622432464/KEGG_AMINOACYL_TRNA_BIOSYNTHESIS.html]

Details for gene set KEGG\_AMINOACYL\_TRNA\_BIOSYNTHESIS[GSEA]

|  || Dataset | 55457\_collapsed\_to\_symbols.cls.cls #OA\_versus\_normal.cls.cls #OA\_versus\_normal\_repos |
| Phenotype | cls.cls#OA\_versus\_normal\_repos |
| Upregulated in class | OA |
| GeneSet | KEGG\_AMINOACYL\_TRNA\_BIOSYNTHESIS |
| Enrichment Score (ES) | 0.22491728 |
| Normalized Enrichment Score (NES) | 0.7999287 |
| Nominal p-value | 0.78281623 |
| FDR q-value | 0.9510638 |
| FWER p-Value | 1.0 |
Table: GSEA Results Summary

  

Fig 1: Enrichment plot: KEGG\_AMINOACYL\_TRNA\_BIOSYNTHESIS      
 Profile of the Running ES Score & Positions of GeneSet Members on the Rank Ordered List

  

| SYMBOL | TITLE | RANK IN GENE LIST | RANK METRIC SCORE | RUNNING ES | CORE ENRICHMENT || 1 | SARS1 | seryl-tRNA synthetase 1 [Source:HGNC Symbol;Acc:HGNC:10537] | 516 | 0.654 | 0.0327 | Yes |
| 2 | EPRS1 | glutamyl-prolyl-tRNA synthetase 1 [Source:HGNC Symbol;Acc:HGNC:3418] | 581 | 0.629 | 0.0978 | Yes |
| 3 | HARS2 | "histidyl-tRNA synthetase 2, mitochondrial [Source:HGNC Symbol;Acc:HGNC:4817]" | 1195 | 0.478 | 0.1033 | Yes |
| 4 | DARS1 | aspartyl-tRNA synthetase 1 [Source:HGNC Symbol;Acc:HGNC:2678] | 1558 | 0.419 | 0.1218 | Yes |
| 5 | IARS2 | "isoleucyl-tRNA synthetase 2, mitochondrial [Source:HGNC Symbol;Acc:HGNC:29685]" | 1685 | 0.401 | 0.1566 | Yes |
| 6 | FARS2 | "phenylalanyl-tRNA synthetase 2, mitochondrial [Source:HGNC Symbol;Acc:HGNC:21062]" | 1686 | 0.401 | 0.2012 | Yes |
| 7 | QARS1 | glutaminyl-tRNA synthetase 1 [Source:HGNC Symbol;Acc:HGNC:9751] | 1913 | 0.371 | 0.2249 | Yes |
| 8 | NARS2 | "asparaginyl-tRNA synthetase 2, mitochondrial [Source:HGNC Symbol;Acc:HGNC:26274]" | 2631 | 0.287 | 0.2011 | No |
| 9 | WARS2 | "tryptophanyl tRNA synthetase 2, mitochondrial [Source:HGNC Symbol;Acc:HGNC:12730]" | 3014 | 0.248 | 0.1991 | No |
| 10 | SARS2 | "seryl-tRNA synthetase 2, mitochondrial [Source:HGNC Symbol;Acc:HGNC:17697]" | 3411 | 0.210 | 0.1917 | No |
| 11 | DARS2 | "aspartyl-tRNA synthetase 2, mitochondrial [Source:HGNC Symbol;Acc:HGNC:25538]" | 4480 | 0.115 | 0.1215 | No |
| 12 | NARS1 | asparaginyl-tRNA synthetase 1 [Source:HGNC Symbol;Acc:HGNC:7643] | 4651 | 0.100 | 0.1194 | No |
| 13 | HARS1 | histidyl-tRNA synthetase 1 [Source:HGNC Symbol;Acc:HGNC:4816] | 5196 | 0.057 | 0.0834 | No |
| 14 | RARS1 | arginyl-tRNA synthetase 1 [Source:HGNC Symbol;Acc:HGNC:9870] | 5469 | 0.035 | 0.0662 | No |
| 15 | TARS2 | "threonyl-tRNA synthetase 2, mitochondrial [Source:HGNC Symbol;Acc:HGNC:30740]" | 5576 | 0.026 | 0.0608 | No |
| 16 | AARS1 | alanyl-tRNA synthetase 1 [Source:HGNC Symbol;Acc:HGNC:20] | 5703 | 0.015 | 0.0528 | No |
| 17 | MARS1 | methionyl-tRNA synthetase 1 [Source:HGNC Symbol;Acc:HGNC:6898] | 6400 | -0.035 | 0.0026 | No |
| 18 | KARS1 | lysyl-tRNA synthetase 1 [Source:HGNC Symbol;Acc:HGNC:6215] | 6837 | -0.066 | -0.0239 | No |
| 19 | LARS2 | "leucyl-tRNA synthetase 2, mitochondrial [Source:HGNC Symbol;Acc:HGNC:17095]" | 7017 | -0.081 | -0.0288 | No |
| 20 | GARS1 | glycyl-tRNA synthetase 1 [Source:HGNC Symbol;Acc:HGNC:4162] | 7069 | -0.085 | -0.0233 | No |
| 21 | CARS2 | "cysteinyl-tRNA synthetase 2, mitochondrial [Source:HGNC Symbol;Acc:HGNC:25695]" | 8958 | -0.228 | -0.1445 | No |
| 22 | CARS1 | cysteinyl-tRNA synthetase 1 [Source:HGNC Symbol;Acc:HGNC:1493] | 9062 | -0.237 | -0.1262 | No |
| 23 | YARS1 | tyrosyl-tRNA synthetase 1 [Source:HGNC Symbol;Acc:HGNC:12840] | 9422 | -0.266 | -0.1244 | No |
| 24 | IARS1 | isoleucyl-tRNA synthetase 1 [Source:HGNC Symbol;Acc:HGNC:5330] | 10154 | -0.337 | -0.1437 | No |
| 25 | WARS1 | tryptophanyl-tRNA synthetase 1 [Source:HGNC Symbol;Acc:HGNC:12729] | 10289 | -0.350 | -0.1153 | No |
| 26 | YARS2 | tyrosyl-tRNA synthetase 2 [Source:HGNC Symbol;Acc:HGNC:24249] | 10299 | -0.350 | -0.0770 | No |
| 27 | VARS1 | valyl-tRNA synthetase 1 [Source:HGNC Symbol;Acc:HGNC:12651] | 11035 | -0.435 | -0.0857 | No |
| 28 | TARS1 | threonyl-tRNA synthetase 1 [Source:HGNC Symbol;Acc:HGNC:11572] | 11357 | -0.480 | -0.0572 | No |
| 29 | LARS1 | leucyl-tRNA synthetase 1 [Source:HGNC Symbol;Acc:HGNC:6512] | 12415 | -0.712 | -0.0601 | No |
| 30 | FARSA | phenylalanyl-tRNA synthetase subunit alpha [Source:HGNC Symbol;Acc:HGNC:3592] | 12714 | -0.878 | 0.0145 | No |
Table: GSEA details [plain text format]

  

Fig 2: KEGG\_AMINOACYL\_TRNA\_BIOSYNTHESIS      
 Blue-Pink O' Gram in the Space of the Analyzed GeneSet

  

Fig 3: KEGG\_AMINOACYL\_TRNA\_BIOSYNTHESIS: Random ES distribution      
 Gene set null distribution of ES for **KEGG\_AMINOACYL\_TRNA\_BIOSYNTHESIS**
